# Patient motivations for seeking online therapy for binge eating disorder

**DOI:** 10.1192/j.eurpsy.2021.948

**Published:** 2021-08-13

**Authors:** T. Holmberg, E. Jensen, J. Bindzus, M. Lichtenstein, K. Tarp

**Affiliations:** 1 Research Unit For Telepsychiatry And Emental Health, Centre for Telepsychiatry, Odense C, Denmark; 2 Centre For Telepsychiatry, Mental Health Services in the Region of Southern Denmark, Odense C, Denmark; 3 Research Unit For Telepsychiatry And E-mental Health, Centre For Telepsychiatry, Mental Health Services in the Region of Southern Denmark, Odense, Denmark

**Keywords:** Internet-based Cognitive Behavioral Therapy, binge eating disorder, qualitative, motivation

## Abstract

**Introduction:**

Binge Eating Disorder (BED) is characterized by repeatedly losing control over eating behavior and consuming large amounts of food within a short period of time. In later years, a growing body of evidence for effectiveness of internet-based Cognitive Behavioral Therapy (iCBT) as treatment for BED has emerged. Regarding the ability to complete a self-help program on the internet, internal self-regulation can be viewed as important.

**Objectives:**

To qualitatively explore patient motivations for seeking therapy for BED according to intrinsic and extrinsic motivation as well as patient reasons for seeking online therapy.

**Methods:**

The research design of this study was qualitatively. The participants were 52 adults suffering from mild to moderate BED. Data consisted of written texts entered by the participants into the online therapy program. The texts addressed the participants’ goals for their treatment course and their motives for seeking online therapy. The texts were analyzed by the means of systematic text condensation.

**Results:**

Pertaining patient motivations for seeking therapy for BED, five main motivations that reached a saturated level in the sample were discovered: wish for control; avoidance of guilt/shame; desire for tools/insights; weight loss; and psychological stress. Participants ranged from one motivational factor to four, no participant had all the motivational factors. Regarding patient reasons for seeking online therapy, the following themes including sub themes were found: online treatment, treatment at home, and flexible treatment.

**Conclusions:**

The results indicate that online therapy for BED may be able to breach some of the barriers there are towards treatment seeking.

